# Processing of Near Real Time Land Surface Temperature and Its Application in Forecasting Forest Fire Danger Conditions

**DOI:** 10.3390/s20040984

**Published:** 2020-02-12

**Authors:** M. Razu Ahmed, Quazi K. Hassan, Masoud Abdollahi, Anil Gupta

**Affiliations:** 1 Department of Geomatics Engineering, Schulich School of Engineering, University of Calgary 2500 University NW, Calgary, AB T2N 1N4, Canada; mohammad.ahmed2@ucalgary.ca (M.R.A.); abdolahi@ucalgary.ca (M.A.); anil.gupta@gov.ab.ca (A.G.); 2 Resource Stewardship Division, Alberta Environment and Parks, 3535 Research Road NW, University Research Park, Calgary, AB T2L 2K8, Canada

**Keywords:** grid data, moderate resolution imaging spectroradiometer (MODIS), natural hazards and disasters, NRT, swath data

## Abstract

Near real time (NRT) remote sensing derived land surface temperature (Ts) data has an utmost importance in various applications of natural hazards and disasters. Space-based instrument MODIS (moderate resolution imaging spectroradiometer) acquired NRT data products of Ts are made available for the users by LANCE (Land, Atmosphere Near real-time Capability) for Earth Observing System (EOS) of NASA (National Aeronautics and Space Administration) free of cost. Such Ts products are swath data with 5 min temporal increments of satellite acquisition, and the average latency is 60-125 min to be available in public domain. The swath data of Ts requires a specialized tool, i.e., HEG (HDF-EOS to GeoTIFF conversion tool) to process and make the data useful for further analysis. However, the file naming convention of the available swath data files in LANCE is not appropriate to download for an area of interest (AOI) to be processed by HEG. In this study, we developed a method/algorithm to overcome such issues in identifying the appropriate swath data files for an AOI that would be able to further processes supported by the HEG. In this case, we used Terra MODIS acquired NRT swath data of Ts, and further applied it to an existing framework of forecasting forest fires (as a case study) for the performance evaluation of our processed Ts. We were successful in selecting appropriate swath data files of Ts for our study area that was further processed by HEG, and finally were able to generate fire danger map in the existing forecasting model. Our proposed method/algorithm could be applied on any swath data product available in LANCE for any location in the world.

## 1. Introduction

Remote sensing data acquired by space-based instruments (i.e., satellite) has an utmost importance in monitoring a wide variety of natural and man-made phenomena on the Earth. To understand the processes of the Earth, such instruments have an ability to acquire and provide timely imagery data for both the atmosphere and land. In which, atmosphere related imagery focuses primarily on characterizing many geophysical dynamics of the troposphere and stratosphere involving clouds, aerosols, temperature, precipitation, lighting, radiation balance, and chemistry [1]. Furthermore, land related imagery products focus primarily on geophysical parameters and processes of the Earth’s surfaces including land and sea surface temperatures, soil moisture, vegetation, and other land covers [2]. Moreover, these remotely sensed data would be able to analyze the processes involved in the Earth’s interior (e.g., tectonics, gravity, and geomagnetism) although originating from below the surface [2]. Nevertheless, geophysical parameters and dynamics of atmosphere and land data acquired by satellites are useful in monitoring hazards and disasters including fires, drought, air quality, ash and smoke plumes, dust storms, floods, severe storms, shipping, and vegetation [3]. For example, near real time (NRT) satellite data have been used to monitor and forecast air quality by estimating air pollutants such as aerosols, carbon monoxide (CO), ozone, nitrogen oxides, and sulfur dioxide in the atmospheric composition [3,4,5,6]. Tracking propagation of such an atmospheric toxic gas like CO due to massive fires is useful to generate early warnings of such pollution spikes to reduce exposure-risk of people to poor air quality by limiting outdoor activities at these times [3]. In addition, biophysical parameters like stress conditions in vegetation and soil moisture have a relation with surface temperature (Ts) [7], and further involve in causing various natural hazards and disasters including agricultural drought and wildland/forest fires.

Several studies demonstrated the effective use of Ts in agricultural drought monitoring and forecasting forest fires by using space-based satellite images. For example, Hazaymeh and Hassan [8] developed an agricultural drought indicator by using remote sensing-derived agricultural drought related variables such as, normalized difference vegetation index (NDVI), normalized difference water index (NDWI), visible and shortwave drought index (VSDI), normalized multiband drought index (NMDI), moisture stress index (MSI), and land surface temperature (Ts). The study used Moderate Resolution Imaging Spectroradiometer (MODIS)-derived 8-day composites of Ts over the growing seasons of 2013–2014 and 2014–2015 in the northwestern part of Jordan, Middle East. In another study by Hu et al. [9] used both 8-day and 16-day composites of MODIS-based Ts over the period of January 2011 to June 2018 for agricultural drought monitoring in the Hetao Plain in Inner Mongolia of Northwest China. Some other studies also used remote sensing based Ts in determining several biophysical parameters of vegetation, such as deciduous phenology [10], understory grass greening stage [11], and surface wetness conditions and growing degree days [12].

In forecasting forest fires, several studies in literature demonstrated the development of remote sensing (RS)-based models that used different MODIS-derived dynamic variables including Ts. For example, Chowdhury and Hassan [13] used MODIS-derived 8-day composites of Ts, NMDI, and NDVI, and daily precipitable water (PW) to forecast at daily scale for the fire seasons of 2009–2011 period. Another study by Abdollahi et al. [14] introduced MODIS-derived daily Ts in their model in addition to the same three MODIS-derived variables (i.e., NMDI, NDVI, and PW) that were used in the model of Chowdhury and Hassan [13] during the fire seasons of 2009–2011. Furthermore, Ahmed et al. [15] improvised the models to forecast at 4-day timescale during fire seasons of 2015–2017 period by using MODIS-derived daily Ts, NDVI, and NDWI variables, and a human-caused static fire danger (SFD) map. In general, in developing forest fires forecasting models to forecast at various timescales (i.e., daily, 4-day, and 8-day), the studies employed standard data products of MODIS for deriving several variables including Ts and various biophysical-related indexes (derived primarily from surface reflectance data).

Although standard data products are internally consistent and well calibrated to support science that served the purposes of those studies; however, the data are made available to public domain after 8–40 h (i.e., not as NRT) upon acquisition of the images by MODIS sensors [16]. Acquiring such a delayed standard data product may not be appropriate for time-critical environmental applications including monitoring and forecasting natural hazards and disasters, where NRT data is the primary requirement. Interestingly, we could acquire NRT data products available in LANCE (Land, Atmosphere Near real-time Capability for Earth Observing System) of NASA (National Aeronautics and Space Administration) at free of cost, which become available in the public domain within 2.5 h of satellite observation [17]. Moreover, MODIS NRT products in LANCE including Ts are available to the public even earlier, where the average latency is 60–125 min [18].

LANCE had been providing MODIS NRT data to support public uses through LANCE-MODIS online hypertext transfer protocol secure (https) sites. For the better availability of MODIS NRT data to the users, LANCE-MODIS has two servers, i.e., NRT3 and NRT4 [19]. Although NRT data products might be downloaded from the both servers (i.e., through https sites); however, NASA recommends to use the main server NRT3. The backup server NRT4 would be useful to download data in the events of non-availability of the main server (i.e., NRT3) for any reason [19]. Nevertheless, for deriving the Ts variable to be used in various time-sensitive applications like natural hazards and disasters, the MODIS NRT in LANCE provides ‘Land Surface Temperature/Emissivity 5-Min L2 1 km’ Swath Data products (i.e., MOD11_L2 from Terra and MYD11_L2 from Aqua) with 5 min temporal increments of satellite acquisition [20].

**Figure 1:**
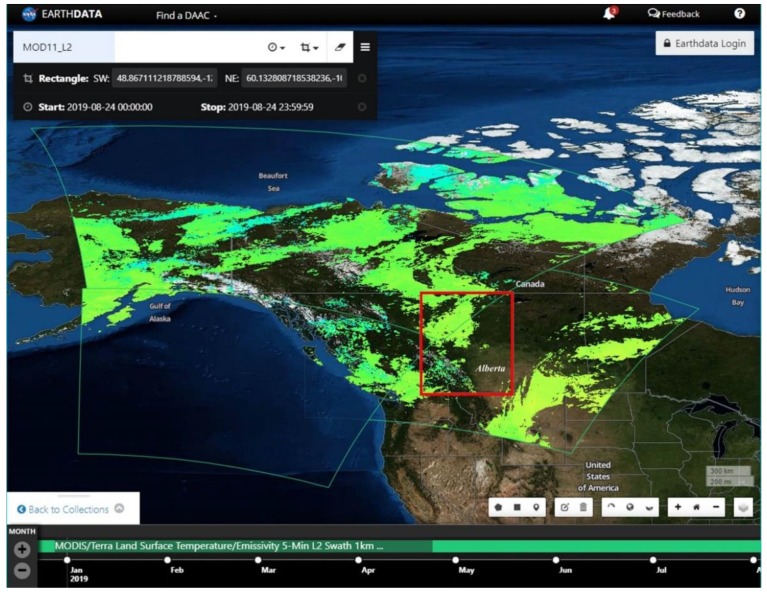
Terra MODIS acquired Land Surface Temperature/Emissivity 5-Min L2 1 km Swath Data showing overlap of three data files of Ts (bounded by green thin curved outlines of the rectangles). The overlapping swath data covering a larger geographic extent for a given AOI of Alberta, Canada (rectangle in red bold outline) that were acquired on 24 August 2019 (source: NASA EARTHDATA). The yellowish green and green to cyan colours in each swath frame were the available land surface temperature (Ts) data, and cloud-contaminated or bad-quality pixels were declared here as ‘no data’.

The MODIS instruments on board the Terra (EOS AM) satellite pass over the equator (i.e., 0° latitude) at approximately 10:30 am and 10:30 pm daily, and Aqua (EOS PM) passes over the equator at approximately 1:30 pm and 1:30 am [21]. Its sun-synchronous orbit allows it to pass over the same area on the Earth in every 24-h period [21]. However, such a repeating sun synchronous orbit acquires the same areas with the same scan angle on the ground every 233 paths (i.e., 16 days). In addition, data might be available covering an area of interest (AOI) on the ground much more frequently (e.g., daily to 3 days), however these are acquired from different overlapping paths with different scan angles (see Figure 1). Nevertheless, the number of overpasses increasing towards the higher latitudes due to overlapping orbits, and the maximum overlapping occurs in the areas close to the poles. Therefore, due to the Earth’s rotation and overlapping overpasses, multiple swath data coverage might be available or required to cover an AOI (even for a very small geographic area) for a day in the higher latitudes. MODIS swath data products are available as each 5-min data that covers an area of 2330 km width and approximately 2100 km along track distance considering each satellite moves at the speed of about 7 km per second. In such cases, there are possibilities of getting multiple swath data files covering a much larger geographical area, or data gaps might be found among the swath coverages for an AOI.

Although the Ts swath data files are made available to the public by LANCE in 60–125 min, it is not possible to identify and download an exact 5-min swath data file for any geographical area on the Earth since there are no coordinate information included in the data file naming convention. We could, of course, download all swath data files for a day, or few swath data files for an estimated few hours’ time period of the overpasses that potentially could cover the extent of an AOI. However, such daily coverage using multiple swath data could have gaps among themselves to cover an AOI or be excessively extended over the globe. NASA provided swath data processing tool HEG (HDF-EOS to GeoTIFF conversion tool) fails to perform reformatting, re-projecting, stitching/mosaicking, and subsetting operations for an AOI in cases any spatial data-gap occurs between swath data files, or the combined coverage is excessively extended over the globe. Therefore, we set our objective in this study to develop a method/algorithm to overcome such issues in identifying the appropriate swath data files by minimizing the number of required files for an AOI that would be able to further processes supported by the HEG. In this case, we planned to use Terra MODIS acquired NRT Swath Data of Ts, and further applied it to an existing framework of forecasting forest fires (as a case study) to understand the performance of the processed Ts.

## 2. Study Area

**Figure 2:**
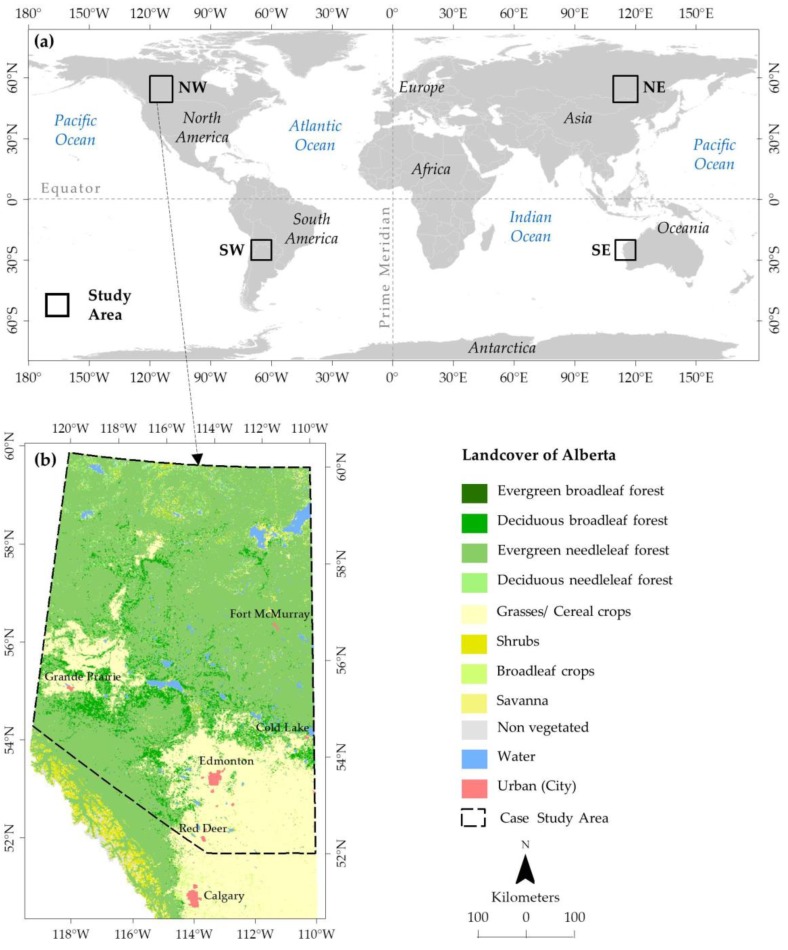
Study areas in the four AOIs located in the NW, NE, SE, and SW hemispheres in the globe (**a**); and processed Ts swath data were used in the forest landcover of Alberta located at the NW AOI to forecast forest fire danger conditions (**b**).

Our study areas were four AOIs in the four quadrants over the globe, such as northwestern (NW), northeastern (NE), southeastern (SE), and southwestern (SW) hemispheres (see Figure 2a) to evaluate the capacity of our proposed algorithm for downloading the MODIS acquired daily swath data of Ts from LANCE server. The MOD11_L2 daily swath data of Ts were further reformat, re-project, stitch/mosaic, and subset by HEG. The geographic coordinates of each AOI in each quadrant are shown in Table 1.

For the evaluation of the daily processed swath data of Ts to be used in an existing framework of forecasting forest fire danger conditions, we used the forested areas in the northern part of Alberta, which is the AOI of the northwestern (NW) hemisphere (see Figure 2b). Among the 21 natural subregions exists in Alberta, we considered four ecological subregions of forest coverage, such as evergreen broadleaf, deciduous broadleaf, evergreen needleleaf, and deciduous needleleaf forests. The subregions in Alberta were categorized based on the differences in vegetation, climate, elevation and physiography [22]. The area experienced about 377 to 535 mm precipitation and −3.6 to 1.1 °C mean annual temperature, and elevated from 162 to 3596 m above msl (mean sea level) [14,15,22].

## 3. Materials

We used LANCE NRT MOD11_L2 data product of land surface temperature (Ts) acquired by Terra MODIS. This product was used to assess the capacity of our proposed algorithm to download and further processing of the daily NRT swath data for any location in the world. For assessing the usability of the processed Ts as a variable to an existing framework of forecast forest fire danger conditions, we additionally used LANCE NRT MOD09GA gridded product for deriving NDVI and NDWI variables that were required for the framework. Furthermore, we used MODIS-derived yearly land cover data product (i.e., MOD12Q1) for the identification of forest areas in the study area; and a human-caused SFD map [14] for generating a final forecast map of forest fire danger conditions. A summary of the data used in this study is shown in Table 2.

## 4. Methods

**Figure 3:**
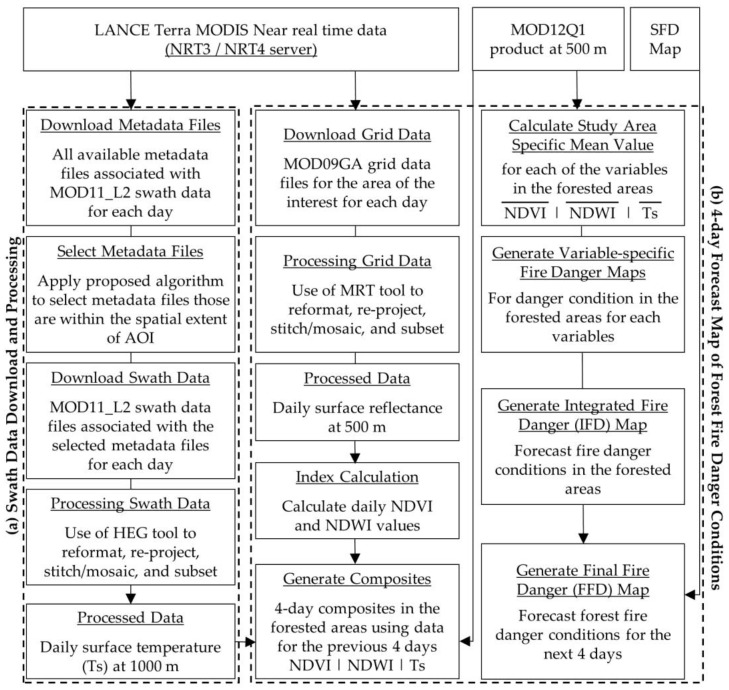
Conceptual diagram of download and processing of Terra MODIS NRT swath data of Ts from LANCE (**a**), and further use of the processed Ts in generating a 4-day forecast map of forest fire danger conditions (**b**).

Figure 3a shows the conceptual diagram to download and processes of Terra MODIS NRT swath data of Ts from the NASA’s LANCE server using our proposed algorithm. Furthermore, the processed Ts data was used in generating a forecast map of forest fire danger conditions using an existing model for the next 4 days [15], as a case study (see Figure 3b). Details on the processing steps are described in the following subsections.

### 4.1. Download Mechanism and Processing of NRT MOD11_L2 Swath Data to Derive Daily Ts

We downloaded all metadata files for a day from LANCE NRT3/NRT4 server, which were associated with the MOD11_L2 data files of the day. Here, each metadata file was about 19 kilobytes in size that were associated with each 5-min data between 00:00 and 23:55; and therefore, the total size of the files was only about 5 megabytes for each AOI. Note that each 5-min swath data file had a corresponding metadata file of the same name with an extension of *.met in the server. The metadata file naming convention for the swath product of a filename MOD11_L2.A2019266.1720.006.NRT. hdf.met (for example) indicated the following:

MOD11_L2—product short name
A2019266—Julian date of acquisition (A-YYYYDDD)
1720—hours and minutes of acquisition (HHMM)
006—collection version
NRT—type of data (i.e., near real time)
hdf—data format (i.e., HDF-EOS)
met—type of file (i.e., metadata for MOD11_L2.A2019266.1720.006.NRT.hdf)

It was not possible to identify any geographic location on the earth from such file naming convention. However, each metadata file contained four-corner coordinates for both longitude (*lon*) and latitude (*lat*) in the lines 183 and 189 respectively that provide the extent of data coverage by each swath data file. Therefore, swath data files were possible to identify for an AOI by using the range of *lon* and *lat* coordinates available in the associated metadata file that intersect with the extent of an AOI. Such a way, we identified only a small number of required metadata files (e.g., two to four metadata files in each day for our study areas that represented swath data covering an AOI). The mathematical expression for such identification of Terra MODIS swath data file with the descending node (i.e., satellite starts data acquisition from the north towards south) is shown as follows (Equation (1)).

(1) $$\left(\left(\begin{array}{l} \left(\left(LRX\leq lon3\right)\;AND\;\left(LRX\geq lon4\right)\right)\;OR\\ \left(\left(ULX\leq lon3\right)\;AND\;\left(ULX\geq lon4\right)\right) \end{array}\right)\;AND\;\left(\begin{array}{l} \left(\left(LRY\leq lat2\right)\;AND\;\left(LRY\geq lat3\right)\right)\;OR\\ \left(\left(ULY\leq lat2\right)\;AND\;\left(ULY\geq lat3\right)\right) \end{array}\right)\right)$$

where, the extent of an AOI was between (ULX, ULY) and (LRX, LRY), the four lon coordinates (i.e., X) in line 183 of a metadata file were lon1, lon2, lon3, and lon4, and the four lat coordinates (i.e., Y) in line 189 were lat1, lat2, lat3, and lat4. Here, lon1, lon2, lon3, and lon4 represented the longitudinal coordinates of the upper-left (UL), upper-right (UR), lower-right (LR), and lower-left (LL) corners of each swath data coverage respectively, and lat1, lat2, lat3, and lat4 represented the latitudinal coordinates of the upper-left (UL), upper-right (UR), lower-right (LR), and lower-left (LL) corners respectively.

Once a limited number of metadata files had been selected for each AOI by using our proposed algorithm, the remaining metadata files were removed. Next, the corresponding MOD11_L2 swath data file (e.g., MOD11_L2.A2019266.1720.006.NRT.hdf) of each selected metadata file (e.g., MOD11_L2.A2019266.1720.006.NRT.hdf.met) was downloaded from NRT3/NRT4 server. The downloaded files were further processed by the HEG tool (a swath data processing tool of NASA) to derive a projected daily surface temperature (Ts) image of an AOI with a spatial resolution size of 1000 m.

### 4.2. Preparation of 4-Day Composite of the Swath Data-Derived Daily Ts

We prepared a 4-day composite of daily Ts images with the planning of using it as a variable in an existing forecast model of forest fire danger conditions [15], as a case study. To prepare such a 4-day composite of Ts image, we used four daily Ts images derived from swath data using our proposed algorithm described in the previous Section 4.1. In generating such a composite image, we stacked the four daily Ts images, and calculated arithmetic mean (average) of the values in the four layers of the stack to derive value for each pixel. In this case, we considered only cloud-free good quality pixels with the assumption of having low forest fire danger conditions in the cloud-contaminated pixels [15]. Furthermore, we resampled the 4-day composite Ts image from 1000 m to 500 m for being consisted with the spatial-resolution of the other variables (i.e., NDVI and NDWI) to be used in the forecast model.

### 4.3. Using the NRT Daily Ts Data and Other NRT Variables to Forecast Forest Fire Danger (A Case Study)

**Figure 4:**
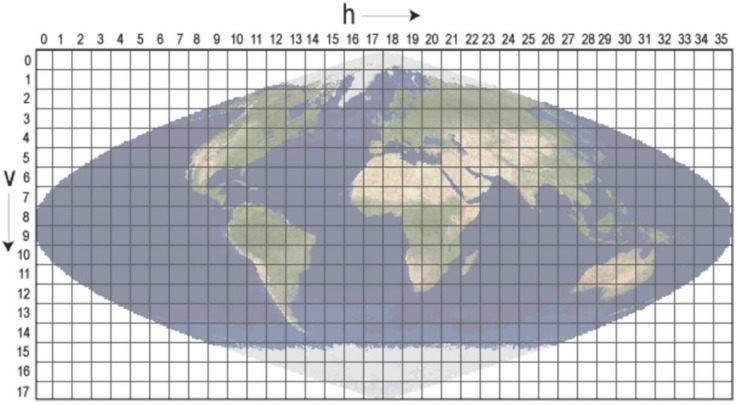
MODIS sinusoidal tile grid consisting of 460 non-overlapping tiles, and each tile covers approximately 10° × 10°.

We followed an existing framework documented in Ahmed et al. [15] to forecast forest fire danger conditions for the next 4 days by using our processed Terra MODIS NRT swath data derived Ts variable (described in the previous Section 4.1). In addition, we used other required variables (i.e., NDVI and NDWI) that were calculated from Terra MODIS NRT gridded data (MOD09GA). Unlike NRT swath data, the MODIS grid data (i.e., MOD09GA) available at LANCE (NRT3/NRT4) were rather simple to select and download for a desired AOI. The MOD09GA grid data file naming convention for a file MOD09GA.A2019266.h11v03.006.NRT.hdf (for example) indicated the followings: *MOD09GA*—product short name; *A2019266*—Julian date of acquisition (A-YYYYDDD); *h11v03*—MODIS sinusoidal grid tile measures at approximately 10° × 10°; *006*—collection version; *NRT*—type of data (i.e., near real time); and *hdf*—data format (i.e., HDF-EOS). Here, it was easy to identify the number of tiles required to cover the spatial extent of a desired AOI, because the coordinates of each sinusoidal grid tile are known, and each tile could be identified by the combination of horizontal (h) and vertical (v) numbers available online (see Figure 4) [23]. Once the tiles are known for an AOI, the MOD09GA grid data files required for an AOI could be selected for download from NRT3/NRT4, because the ‘h’ and ‘v’ information are included in the file names. Moreover, a downloaded daily grid data (i.e., MOD09GA) for an AOI could be further processed by the MODIS Reprojection Tool (MRT, a NASA tool) for deriving a daily surface reflectance image (includes bands 1 to 7) of an AOI with a spatial resolution of 500 m. Nevertheless, the processes of generating a forest fire danger map using the existing framework are briefly described below.

Firstly, we downloaded daily MOD09GA grid data tiles from LANCE NRT3 server for our NW study area using 10 to 12 and 03 for h and v respectively (i.e., h10v03, h11v03 and h12v03). Once these three tiles were downloaded, we used the MRT tool to derive a projected daily surface reflectance image at 500 m spatial resolution by mosaic and subset operations. Next, three bands, such as band 1 (*red*), band 2 (near infrared, *nir*), and band 7 (shortwave infrared 3, *swir*3) were used to calculate daily NDVI and NDWI images for the study area. Such daily NDVI and NDWI images were generated for the previous four days. Furthermore, 4-day composite images of NDVI and NDWI variables were generated with the daily NDVI and NDWI images respectively created for the previous four days. In generating the composite images, we calculated the variable-specific maximum values for each pixel in the images in each composite during the composition period. Furthermore, we performed subset operation on these two composite images (i.e., NDVI and NDWI) and the 4-day composite of Ts (generated in the previous Section 4.2). For the subset, we used locations of forest classes available in MOD12Q1 thematic data and the provincial boundary of Alberta to derive data of the calculated variables in forested regions only.

Secondly, we generated thematic maps of variable-specific forest fire danger conditions for the next 4 days. In preparing those maps, we calculated the variable-specific average value for the study area (i.e., the ‘Global Mean’ of each Ts, NDVI and NDWI composite for the previous 4 days). Once the global mean values were calculated for each variable, we prepared variable-specific thematic maps containing two fire danger (FD) classes (i.e., ‘high’ and ‘low’) over the forested areas using the criteria documented in Ahmed et al. [15].

Finally, we prepared an integrated fire danger (IFD) map with the variable-specific danger conditions by considering same weightage for each variable. The resulted IFD map was consisted of four FD classes, such as very high (VH), high (H), moderate (M), and low (L) depending on the total number of variables occurred in the ‘high’ danger class. Next, we generated a final forest fire danger (FFD) map to forecast forest fire danger conditions for the next four days. To derive the FFD map, we additionally integrated a static fire danger (SFD) map with ‘high’ and ‘low’ danger classes describing the influence of human-caused ignition source (adopted from Abdollahi et al. [14]). During the operation, four FD classes in the IFD map were reassigned to four classes in the FFD map depending on the danger conditions available in the SFD map. For example, we assigned a higher danger class (i.e., H to VH, M to H, and L to M), where the corresponding pixels in the SFD map demonstrated ‘high’ danger class. However, categories remained unchanged in case of ‘low’ danger class.

## 5. Results and Discussion

### 5.1. Daily Ts Image from MOD11_L2 Swath Data

**Figure 5:**
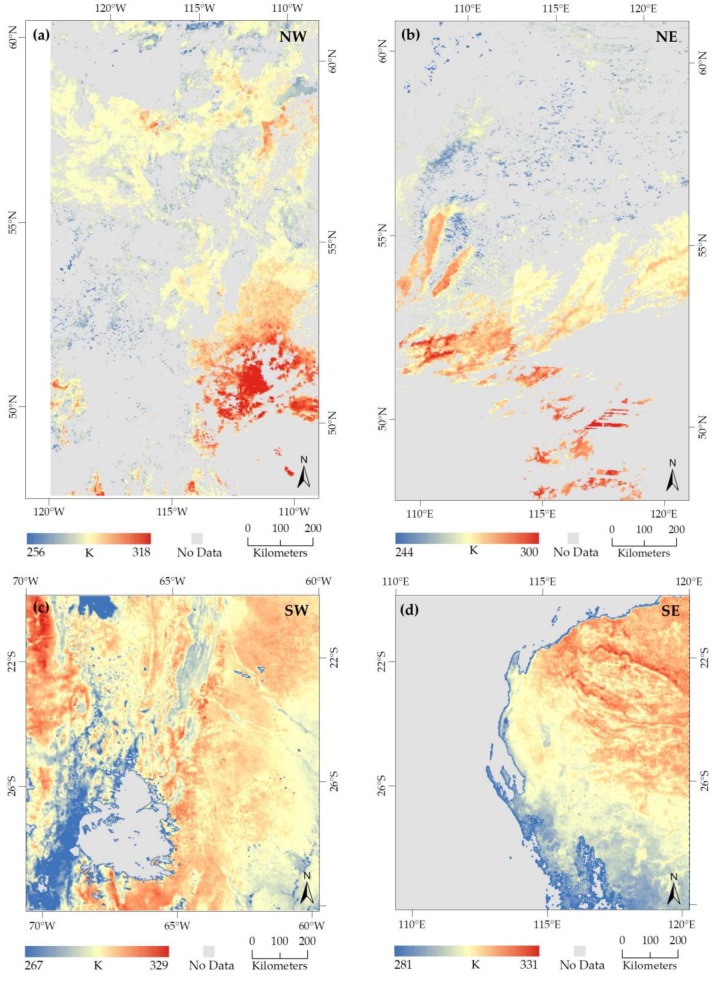
Daily Ts images in Kelvin (K) for the four different AOIs. Each Ts image was derived from the mosaic, reprojection, and subset operations on multiple swath data (i.e., MOD11_L2) for a day in 2019. For example, (**a**) northwestern hemisphere (NW): DOY 236; (**b**) northeastern hemisphere (NE): DOY 275; (**c**) southwestern hemisphere (SW): DOY 273; and (**d**) southeastern hemisphere (SE): DOY 275. Only cloud-free good pixels are shown here and remaining declared as ‘no data’.

Daily Ts image was generated for each AOI of our study area from multiple daily MOD11_L2 swath data using our proposed algorithm. For each AOI, we generated four different daily Ts images. Ts images were generated for the following days: (i) 24–27 August 2019 (day of year, DOY 236 to 239) for NW; (ii) 29 September–2 October 2019 (DOY 272 to 275) for NE; (iii) 30 September–3 October 2019 (DOY 273 to 276) for SW; and (iv) 29 September–2 October 2019 (DOY 272 to 275) for SE. Among the generated daily Ts images for NW, NE, SW, and SE locations, we are presenting daily Ts images of 24 August 2019 (Figure 5a), 2 October 2019 (Figure 5b), 30 September 2019 (Figure 5c), and 2 October 2019 (Figure 5d) respectively, as example outputs. The daily Ts images located in the northern hemisphere (NW and NE coverages) were having much more cloud-contaminated pixels (declared as no-data in Figure 5) in the land areas. Although, these dates were considered under the window of forest fires season; however, fires are less pronounced around such late season in northern latitudes, which is probably because of the presence of excessive cloud coverage.

### 5.2. Four-Day Composite of Ts

**Figure 6:**
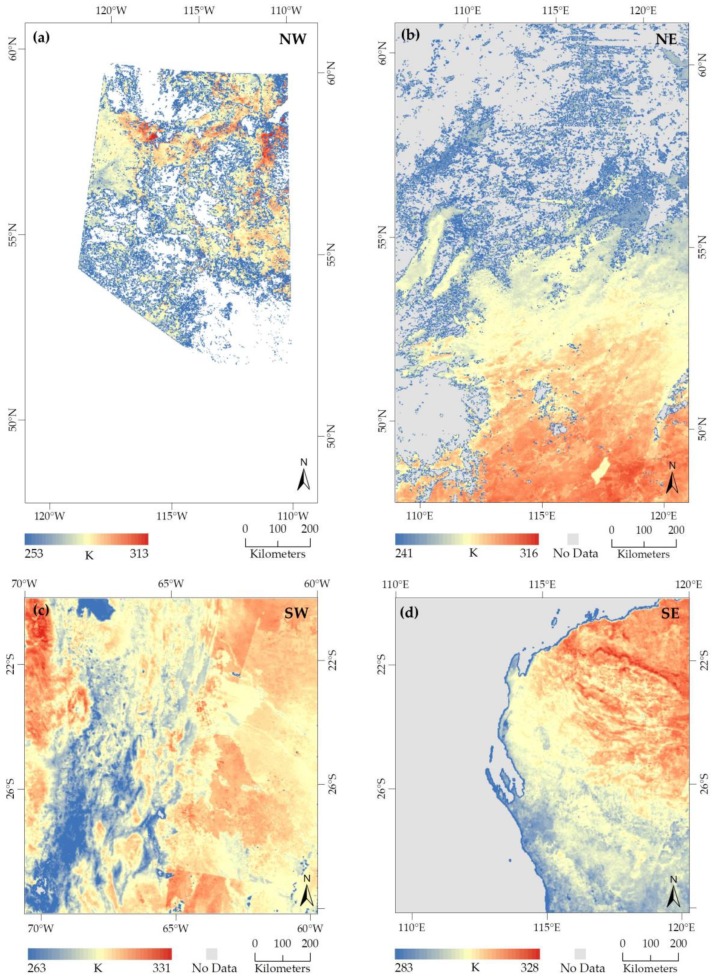
4-day composites of Ts derived from four daily Ts images of each AOI. The dates of daily Ts images spanning over a four-day composition period were: (**a**) NW: DOY 236 to 239 (24–27 August 2019); (**b**) NE: DOY 272 to 275 (29 September–2 October 2019); (**c**) SW: DOY 273 to 276 (30 September–3 October 2019); and (**d**) SE: DOY 272 to 275 (29 September–2 October 2019).

The 4-day Ts composite images were prepared for each of the AOI that comprised of the daily Ts images derived from NRT swath data presented in the previous subsection (see Section 5.1). The 4-day composites of Ts for NW, NE, SW, and SE locations were comprised of daily Ts images of DOY 236 to 239 (see Figure 6a), 272 to 275 (see Figure 6b), 273 to 276 (see Figure 6c), and 272 to 275 (see Figure 6d) in 2019 respectively. Since our objective was to prepare an FFD map only for the forested areas, we generated a cropped 4-day composite of Ts image (see Figure 6a) covering the vegetation in our NW study area. The remaining areas were declared as ‘no data’ and assigned as transparent (white areas in Figure 6a). Note that we did not apply any vegetation coverage or administrative boundary layer for the remaining AOIs (i.e., NE, SW and SE), and thus the 4-day Ts composite images of those areas were having data for the entire AOI (see Figure 6b–d). However, ‘no data’ areas in those images (see Figure 6b–d) were due to the presence of cloud-contaminant pixels or not good-quality pixels.

### 5.3. Daily NRT Swath Data-derived Ts and Other NRT Variables to Forecast Forest Fire Danger Conditions

**Figure 7:**
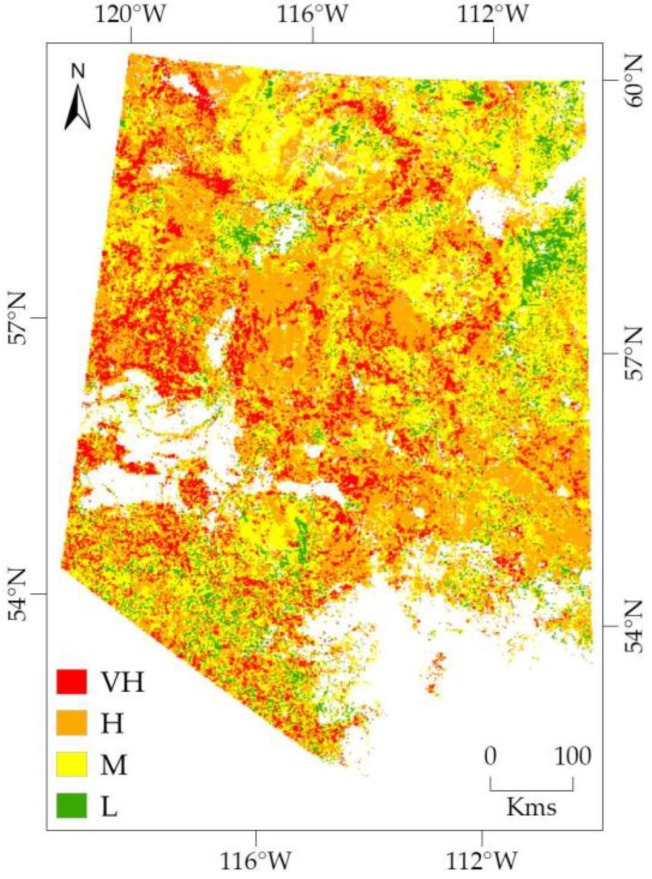
An FFD map was generated using NRT swath data of Ts variable that was derived by applying our proposed algorithm, and NRT gridded data for calculating NDVI and NDWI variables.

The existing 4-day scale forecasting model of forest fire danger conditions used four variables including three dynamic variables such as, Ts, NDVI and NDWI, and a static variable, i.e., an SFD map. In our study, we used the dynamic variables that were derived from NRT MODIS data, such as, NRT swath data (MOD11_L2) for Ts and NRT gridded data (MOD09GA) for calculating NDVI and NDWI. We were able to produce an FFD map at NRT (on 28 August 2019) by using 4-day composites of each dynamic variable acquired from an NRT source for a period of previous four days (i.e., 24–27 August 2019) to forecast for the next four days (i.e., 28–31 August 2019). The FFD map of the forested areas in our NW study location were having four FD categories, such as VH, H, M, and L (see Figure 7). Note that we could not validate the FFD map since no NRT fire occurrence data was available. Furthermore, the existing model that we used to forecast forest fire danger conditions by integrating our proposed algorithm of deriving Ts image from NRT swath data had already been validated and documented a very good accuracy [15].

In the scope of this manuscript, it was not possible to provide a detailed validation of the proposed NRT model. Although there were two potential sources of reference data available for such a detailed validation; however, we could not use those for the following two reasons. Firstly, the most reliable database on fire occurrences in Canada is known as the Canadian National Fire Database (CNFDB). Such data is synthesized by the Government of Canada, and available for the public to download (see https://cwfis.cfs.nrcan.gc.ca/datamart for details; accessed on 7 February 2020). However, the database usually includes the historical fire occurrences until one to three years back from the on-going or recent fire season. Therefore, it was not possible to derive any NRT fire occurrence data that would be useful for detailed validation of our NRT model generated FFD maps. Secondly, another possibility of validating the FFD maps was to use the active fire spot images of the MODIS and VIIRS (Visible Infrared Imaging Radiometer Suite). NASA’s fire information for resource management system (FIRMS) distributes such NRT active fire data within 3 h of satellite observation (see https://earthdata.nasa.gov/earth-observation-data/near-real-time/firms for details; accessed on 7 February 2020). However, validating with such data products is having some limitations, e.g., the confidence value of the products varies from 0 to 100%, and the data might produce false alarms (i.e., actual forest fires do not occur in the areas) over bright/reflective surfaces [15,24,25,26,27,28]. Hence, using a dataset with such a variable confidence level and possibility of having false alarms was not appropriate to validate the FFD maps generated by our NRT model.

It would be worth noting that the MODIS-derived Ts data and other required variables to forecast forest fire danger conditions were successfully used in previous studies [13,14,15]. Despite their reasonable performances against ground observed data, they did not have the capacity to be used as operational prototypes. This was due to the fact that they employed historical remote sensing data, and that made them possible to perform detailed validations mostly based on the historical CNFDB. In this study, we used NRT data to make it an operational model as discussed before. The framework of this NRT model was very much similar to the validated and published framework of Ahmed et al. [15]. The major differences were that we proposed methods/algorithms of using the MODIS-acquired NRT swath and grid data to develop an operational NRT model, where FFD maps were generated as a case study. Besides, we plan to implement this proposed NRT model in the upcoming fire season of 2020 spanning between May and September in Alberta, Canada, which would further facilitate the detailed validation in future.

In developing such operational model based on NRT MODIS-derived variables, the major challenge was to use NRT swath data of Ts. Therefore, we presented the outcomes of derived daily Ts images from NRT swath data by using our proposed algorithm. Furthermore, we showed the usability of the derived NRT Ts image in forecasting forest fire danger conditions, as a case study, for the NW study area. Note that we did not generate FFD maps for other three locations (i.e., NE, SE, and SW) in this study. However, we generated daily Ts images (see Section 5.1) and Ts composites (see Section 5.2) for those three locations to evaluate the effectiveness of the proposed algorithm.

## 6. Conclusions

In this study, we presented an algorithm/method that would be able to identify appropriate MODIS NRT swath data files required for an AOI by minimizing the number of files supported by the HEG tool to process it. In this case, we used Terra MODIS acquired NRT swath data of Ts, and further applied it to an existing framework of forecasting forest fires to assess performance of the processed Ts. In addition, we used Terra MODIS acquired NRT gridded data for calculating other variables (i.e., NDVI and NDWI) that were required for the existing model. Using those Terra MODIS acquired NRT data in the existing forest fires forecasting model, we were successful to generate a final danger map (FFD) to forecast for the next four days. Outcomes in this study showed a promising future in developing remote sensing based NRT forecasting models not only for forest fires but also for other natural hazards and disasters related to Ts. Theoretically, other remotely sensed products in LANCE available as Terra MODIS NRT swath data could be downloaded, processed, and applied in other applications by using our proposed method. Moreover, Aqua MODIS NRT swath data could be downloaded and processed by using the proposed method that would be useful in various applications. However, we suggest evaluating the method before final adoption for any operational purposes. This proposed NRT model to forecast forest fire danger conditions would be helpful for the fire managers to initiate immediate protocols to evaluate and manage large fire occurrences. Besides, the local governments, industries including oil sand, local and indigenous communities, etc. located in the heart of boreal forested areas would benefit by becoming resilient to forest fires as a result of using this model.

## Figures and Tables

**Table 1:** Coordinates of the extents of four AOIs that were used to evaluate our proposed algorithm in selecting appropriate swath data files.

Area of Interest	ULX *	ULY *	LRX *	LRY *
NW (northwestern hemisphere)	−120	61	−109	48
NE (northeastern hemisphere)	109	61	121	48
SE (southeastern hemisphere)	110	−20	120	−30
SW (southwestern hemisphere)	−70	−20	−60	−30

* ULX: upper left X; ULY: upper left Y; LRX: lower right X; and LRY: lower right Y coordinates.

**Table 2:** Brief description of the datasets used in this study.

Data Product	Satellite/Sensor	Source	Description	Purpose of Use
MOD11_L2 (raster)	Terra MODIS	NASA’s Land, Atmosphere Near-real-time Capability for EOS (LANCE): NRT3/NRT4	Land Surface Temperature and Emissivity 5-min L2 Swath imagery (v006) at 1 km spatial resolution.	Preparing daily Ts image to generate composite for an intended period.
MOD09GA (raster)	Terra MODIS		Surface Reflectance Daily L2G Global imagery (v006) Bands 1 to 7 at 500 m spatial resolution.	Calculating daily NDVI and NDWI images.
MOD12Q1 (raster)	Terra+Aqua MODIS	NASA’s Earthdata	Land Cover Type Yearly L3 Global SIN Grid at 500 m spatial resolution.	To identify intended forest vegetation coverage.
SFD map (raster)	-	Abdollahi et al. [14]	SFD derived from 500 m buffered road network of Alberta.	As a variable in generating the final FD map.
Boundary (vector)	-	Government of Alberta	Provincial boundary of Alberta	For defining the study area.
